# Supplementary material for: Evolution of AF6-RAS association and its implications in mixed-lineage leukemia
Source: Nat Commun. 2017 Oct 23;8:1099. doi: 10.1038/s41467-017-01326-5 (PMC5653649; doi:10.1038/s41467-017-01326-5)
Supplement: Supplementary file 1 — Supplementary Information [file 41467_2017_1326_MOESM1_ESM.pdf]

Supplementary Figures

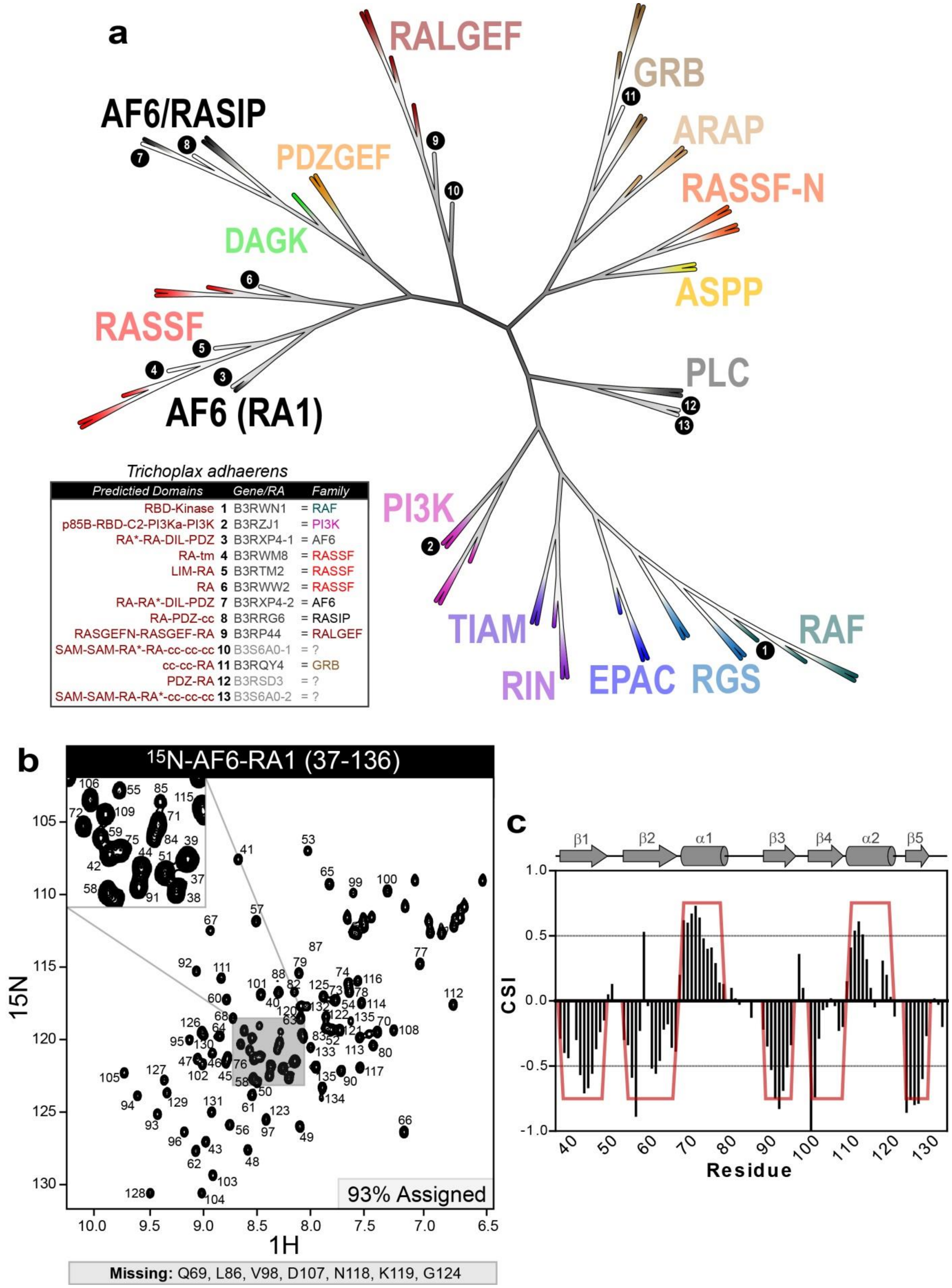

Supplementary Fig. 1. Proteins containing RAS binding domains in the basal metazoan *Trichoplax adhaerens* show correlating domain organizations and strong sequence similarity to their human orthologues. (**a**) To identify the most evolutionarily conserved RAS effectors, amino acid sequences from all 52 RBD domains we could identify in the human proteome were aligned together with 13 RBD domains in proteins encoded by 11 genes in *T. adhaerens*. Boxed; predicted domain organization for RAS effectors in *T. adhaerens* (red; SMART, PFAM), gene names, and their proposed human orthologues based on RBD domain sequence conservation and domain mapping. (**b**) 2D $^{1}H/^{15}N$-HSQC spectra of AF6 RA1 domain, residues 37-136. Backbone assignment was completed for 93% of residues using standard triple resonance experiments (HNCACB, CBCACONH) on $^{15}N/^{13}C$ protein (7 residues missing listed at bottom). (**c**) CSI versus residue number for the RA1 domain of AF6. Four positive CSI values indicate α-helix; four negative values indicate β-strand. Resulting secondary structure arrangement depicted at top is prototypical of RBD domains.

a

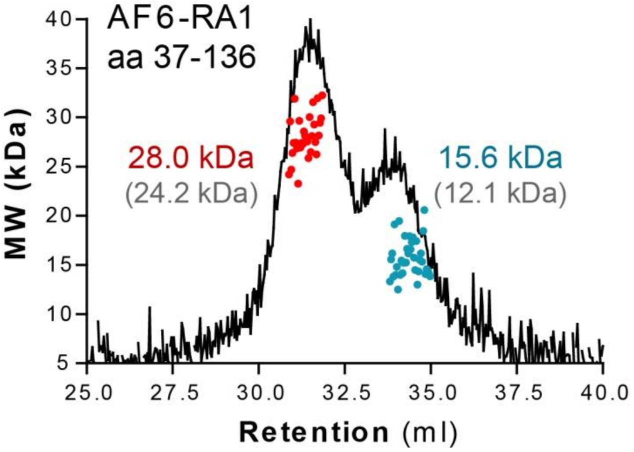

b

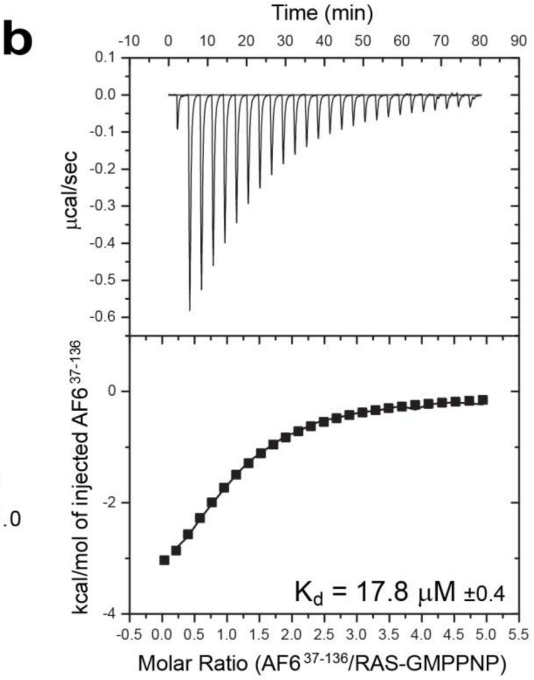

c

**Data Collection**	
Space group	$P\,2_12_12_1$
PDB code	6AMB
Cell dimensions	
a, b, c (Å)	48.2, 57.2, 73.3
α, β, γ (°)	90, 90, 90
Resolution (Å)	40.3-2.499 (2.59-2.5)
$R_{sym}(I)$ (%)	0.097 (0.557)
$I/\sigma(I)$	12.0 (3.4)
Completeness (%)	99.6 (96.0)
Redundancy	6.9 (6.6)
**Refinement**	
R_{work}	0.218
R_{free}	0.266
No. atoms	
Macromolecules	1973
Ligands	33
Water	10
B-factors (Å^2)	
Macromolecules	54,6
Ligands	38.5
Solvent	45.2
Ramachandran statistics	
Most favorable regions (%)	98
Allowed regions (%)	2
Disallowed regions (%)	0
R.m.s. deviations	
Bond lengths (Å)	0.004
Bond angles (°)	0.97

d

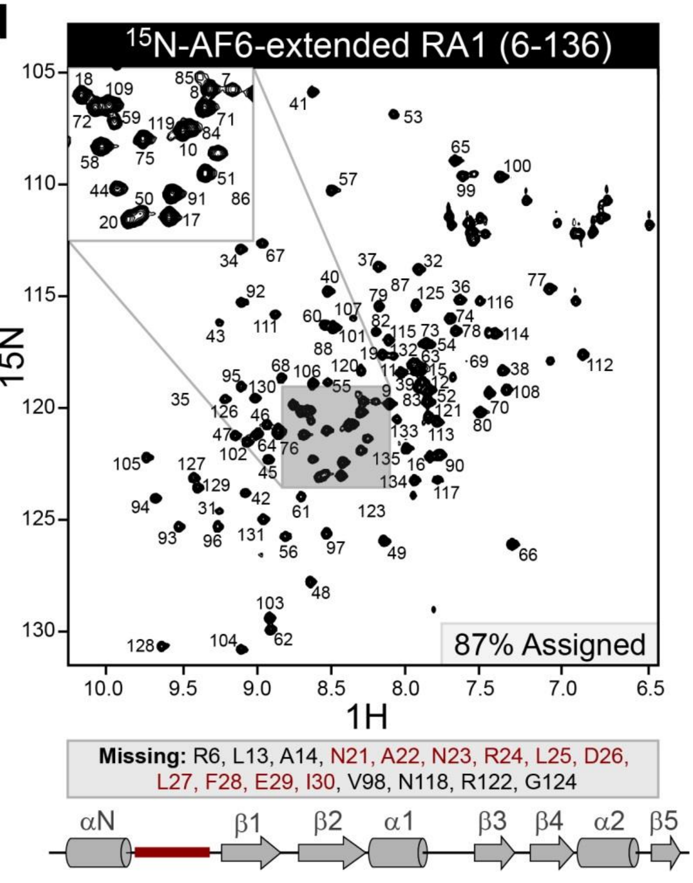

Supplementary Fig. 2. Biochemical properties of the core AF6 RA1 domain, backbone assignment for the N-terminally extended AF6 RA1 domain, and structure statistics for the X-Ray crystal structure of RA1 complexed with RAS-GMPPNP. (**a**) Size-exclusion chromatography in line with multi-angle light scattering (MALS) to determine the molecular weight of purified AF6 RA1 domain at 4°C. MALS-determined molecular weights of the larger peak (red) were consistent with a dimer, while the smaller peak (blue) was consistent with a monomer. (**b**) ITC shows a weak association between the AF6 RA1 domain (residues 37-136) and RAS-GMPPNP. Purified RA1 domain was injected into purified RAS-GMPPNP with a resultant dissociation constant (K_d) of 17.8 μM, an order of magnitude weaker than most RAS-effector interactions. (**c**) Data collection and refinement statistics for the co-crystal structure of AF6 RA1 domain and RAS-GMPPNP (PDB code 6AMB). Values within parentheses refer to the highest resolution shell. (**d**) 2D $^{1}H/^{15}N$-HSQC spectra of an extended AF6 RA1 domain, residues 6-136. Backbone assignment was completed for 87% of residues using standard triple resonance experiments (HNCACB, CBCACONH) on $^{15}N/^{13}C$ protein. The majority of unassigned residues (bottom) are in the loop region following the αN helix (red).

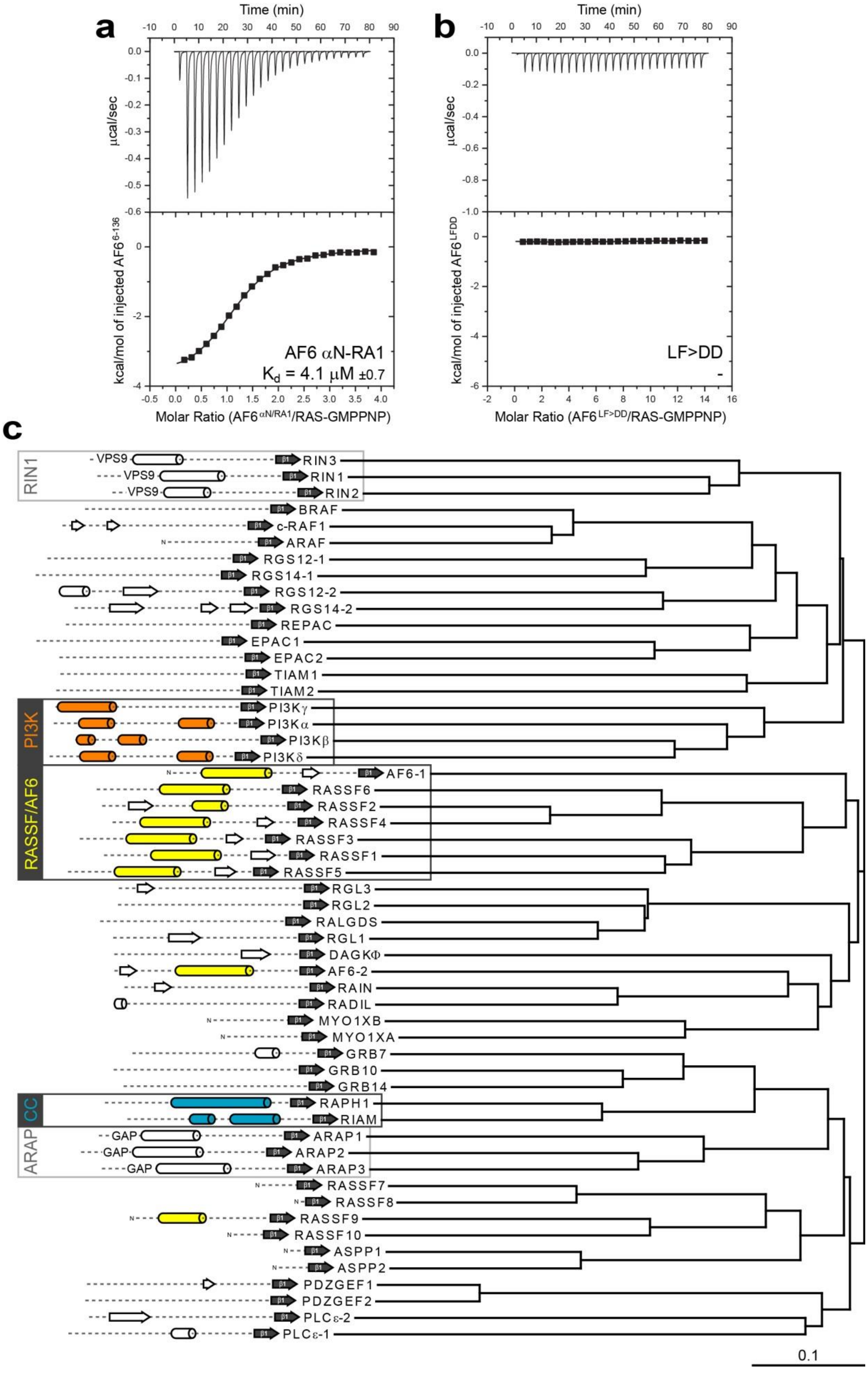
a
b
c
Time (min)
µcal/sec
kcal/mol of injected AF6^6-136
Molar Ratio (AF6^αN/RA1/RAS-GMPPNP)
AF6 αN-RA1
K_d = 4.1 µM ±0.7
kcal/mol of injected AF6^LFDD
Molar Ratio (AF6^LF>DD/RAS-GMPPNP)
LF>DD
-
RIN1
PI3K
RASSF/AF6
CC
ARAP
VPS9
GAP
RIN3
RIN1
RIN2
BRAF
c-RAF1
ARAF
RGS12-1
RGS14-1
RGS12-2
RGS14-2
REPAC
EPAC1
EPAC2
TIAM1
TIAM2
PI3Kγ
PI3Kα
PI3Kβ
PI3Kδ
AF6-1
RASSF6
RASSF2
RASSF4
RASSF3
RASSF1
RASSF5
RGL3
RGL2
RALGDS
RGL1
DAGKΦ
AF6-2
RAIN
RADIL
MYO1XB
MYO1XA
GRB7
GRB10
GRB14
RAPH1
RIAM
ARAP1
ARAP2
ARAP3
RASSF7
RASSF8
RASSF9
RASSF10
ASPP1
ASPP2
PDZGEF1
PDZGEF2
PLCε-2
PLCε-1
0.1

Supplementary Fig. 3. An RA1 domain with the extended helix demonstrates significantly tighter binding to RAS-GMPPNP, and only a small subset of RAS binding domains interact with RAS in this manner. (**a**) ITC substantiates the contribution of the αN helix to the AF6 RA1 domain interaction with RAS. An extended RA1 domain (residues 6-136) was titrated into RAS-GMPPMP with a resultant dissociation constant (K_d) of 4.1 μM, 4.5-fold tighter than the core RA1 domain alone. (**b**) Mutation of the Leu-Phe motif completely disrupts AF6 binding to RAS, as measured by ITC. An L27D/F28D double mutant of the extended AF6 RA1 domain was titrated into RAS-GMPPNP, with no discernible heats of interaction. (**C**) RA domains from the effector proteins RASSF(1-6) and AF6 are the only RAS-interacting domains with the αN helix. Amino acid alignment was performed using core RBD domains from all RAS effectors in the human proteome (determined using secondary structure predictions for the common ubiquitin superfold structure ββαββαβ; JPRED) as well as 50 residues upstream of β1 to account for presence of an αN helix. Alignment tree reveals families of conserved effectors, and secondary structure predictions of the 50 upstream residues are shown to the left. The RIN1 and ARAP effectors (white outline) have helices N-terminal to β1, but these are components of the preceding domains (VPS9 and GAP, respectively). PI3K RBD domains are predicted to have short helices (orange), which are evident in the published PI3K-RAS structure (PDB 1HE8) but do not contact the RAS G domain. RAPH1 and RAIM have N-terminal helices (blue) that function as coiled-coils, analogous to the related Lamellipodin (PDB 4GMV). The only remaining RAS binding domains with predicted αN helices are the RA1 domain of AF6 and the RA domains of RASSF effectors 1-6 (yellow), which cluster together denoting a high level of sequence conservation.

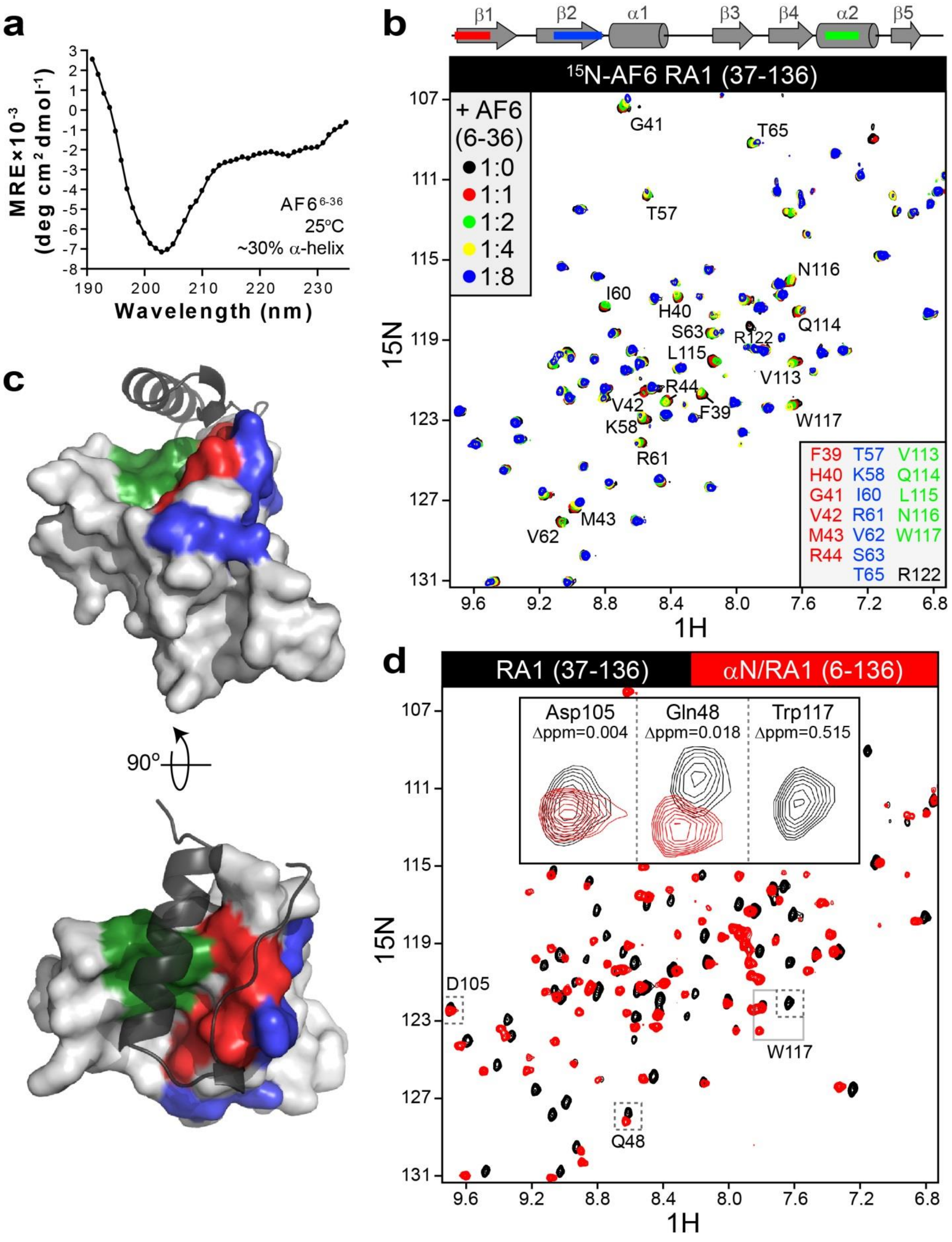
a
MRE×10⁻³ (deg cm² dmol⁻¹)
AF6 6-36
25°C
~30% α-helix
Wavelength (nm)
b
β1 β2 α1 β3 β4 α2 β5
15N-AF6 RA1 (37-136)
+ AF6 (6-36)
1:0
1:1
1:2
1:4
1:8
G41 T65 T57 N116 I60 H40 S63 R122 Q114 L115 V113 R44 V42 F39 W117 K58 R61 M43 V62
F39 H40 G41 V42 M43 R44
T57 K58 I60 R61 V62 S63 T65
V113 Q114 L115 N116 W117
R122
15N
1H
c
90°
d
RA1 (37-136)
αN/RA1 (6-136)
Asp105 Δppm=0.004
Gln48 Δppm=0.018
Trp117 Δppm=0.515
D105
W117
Q48
15N
1H

Supplementary Fig. 4. Interface between the αN helix and core RA domain. (**a**) Far-UV CD spectroscopy demonstrates AF6 residues 6-36 have propensity to form an α helix in solution. At 25°C, the purified 30 residue protein is estimated to be 30% α-helical. (**b**) $^{1}H/^{15}N$-HSQC spectra showing chemical shift broadening of AF6 RA1 domain upon titration of AF6 residues 6-36. Increasing molar ratios (top left) of the short helical segment were added to the RA domain alone (black), starting at 1:1 (red) and going up to 1:8 (blue). 19 resonances showed significant exchange broadening, listed at bottom right. The broadened residues are derived from 3 sequential amino acid sequences in the AF6 RA1 domain (coloured red, blue and green), overlaid in the domain diagram at top as positioned in β1, β2 and α3. (**c**) Position of surface residues broadened by titration of AF6 amino acids 6-36 in the AF6 RA1 domain structure. Red, blue and green colours correspond to sequential broadened regions identified in **b**. The location of these residues is consistent with our model of the αN helix (black) based on structural alignment with the RASSF5-RAS structure (Fig. 2b). (**d**) Overlay of $^{1}H/^{15}N$-HSQC spectra from AF6 core RA1 domain (37-136) and the extended RA1 domain incorporating the αN helix (6-136). Combined chemical shift perturbation (Δppm) of backbone ^{15}N and ^{1}H resonances was calculated using the equation: $\Delta ppm = \sqrt{}\Delta^{1}H + 0.14\Delta^{15}N$. Boxed, three resonances with calculated Δppm representative of negligible perturbations (Asp105 and Gln48) or significant perturbation (Trp117).

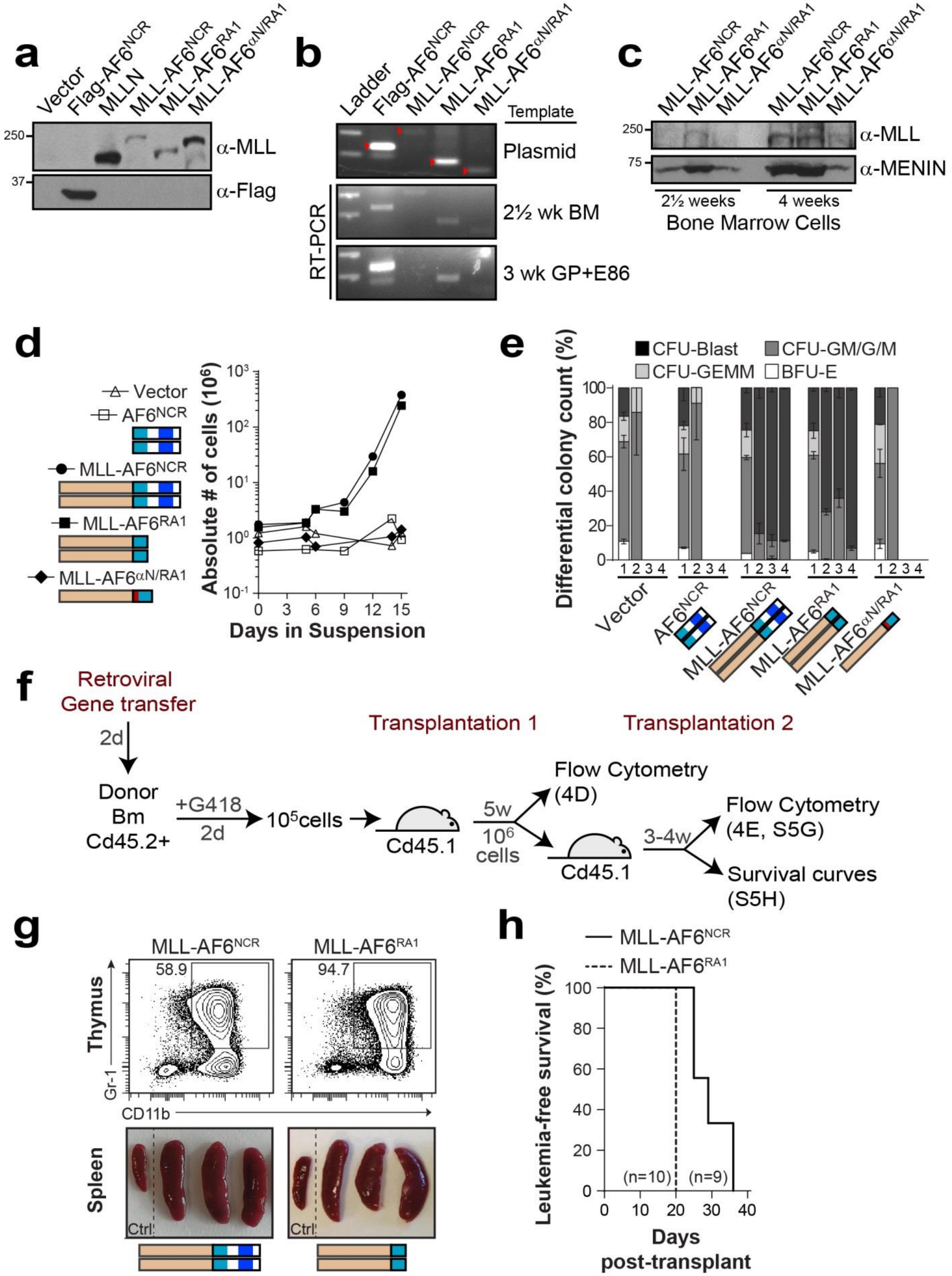

a
Vector
Flag-AF6NCR
MLLN
MLL-AF6NCR
MLL-AF6RA1
MLL-AF6αN/RA1
250
37
α-MLL
α-Flag
b
Ladder
Flag-AF6NCR
MLL-AF6NCR
MLL-AF6RA1
MLL-AF6αN/RA1
Template
Plasmid
2½ wk BM
3 wk GP+E86
RT-PCR
c
MLL-AF6NCR
MLL-AF6RA1
MLL-AF6αN/RA1
MLL-AF6NCR
MLL-AF6RA1
MLL-AF6αN/RA1
250
75
α-MLL
α-MENIN
2½ weeks
4 weeks
Bone Marrow Cells
d
Vector
AF6NCR
MLL-AF6NCR
MLL-AF6RA1
MLL-AF6αN/RA1
Absolute # of cells (10^6)
Days in Suspension
e
CFU-Blast
CFU-GM/G/M
CFU-GEMM
BFU-E
Differential colony count (%)
Vector
AF6NCR
MLL-AF6NCR
MLL-AF6RA1
MLL-AF6αN/RA1
f
Retroviral
Gene transfer
2d
Donor
Bm
Cd45.2+
+G418
2d
10^5 cells
Transplantation 1
Cd45.1
5w
10^6
cells
Flow Cytometry
(4D)
Transplantation 2
Cd45.1
3-4w
Flow Cytometry
(4E, S5G)
Survival curves
(S5H)
g
MLL-AF6NCR
MLL-AF6RA1
58.9
94.7
Thymus
Gr-1
CD11b
Spleen
Ctrl
h
MLL-AF6NCR
MLL-AF6RA1
Leukemia-free survival (%)
(n=10)
(n=9)
Days
post-transplant

Supplementary Fig. 5. Insertion of the αN helix between MLL and the AF6 RA1 domain disrupts myeloid immortalization and blocks the oncogenic potential of MLL in mice. (**a**) Transient expression of MLL fusion proteins in HEK 293 cells. The N-terminal half of MLL (1-1395) was fused to AF6 residues 35-348 (MLL-AF6^{NCR}), 37-136 (RA1 domain) or 6-136 (αN-extended RA1) in pMSCVneo for retrovirus production. FLAG-tagged AF6 residues 35-314 (AF6^{NCR} in the absence of MLL) was used as a negative control. (**b**) Confirming expression of MLL fusion proteins by RT-PCR. Primers were designed to specifically detect *MLL* fusion to sequences encoding AF6^{NCR}, AF6^{RA1} or AF6$^{\alpha N\text{-}RA1}$. RT-PCR was performed on cDNA from whole RNA extracts taken from mouse BM or GP+E86 cells, either 2½ or 3 weeks post-transduction, respectively. (**c**) Western blot to detect expression of MLL fusion proteins in transduced bone marrow cells maintained in primary culture. anti-MLL (top) reveals high levels of MLL-AF6^{NCR} and MLL-AF6^{RA1} 4 weeks post-transduction. Increased levels of the MLL binding partner MENIN are also observed (bottom). (**d**) Cumulative growth curve of transduced cells in primary cultures. Hematopoietic precursor cells (Lineage-neagative) expressing MLL-AF6^{NCR} and MLL-AF6 RA1 domain (37-136) showed comparable exponential growth. MLL fused to αN-extended RA1 (6-136) was undistinguishable from negative controls (vector alone and FLAG-AF6^{NCR}). (**e**) Distribution of myeloid populations in colonies derived from primary hematopoietic progenitors immortalized by retroviral constructs. Bars represent mean ± SD for the distinct populations. In contrast to blast colonies, CFU-GEMM decline in serial replating. (**f**) Strategy for serial transplantation of MLL fusion expressing cells into mice. (**g**) MLL-AF6^{NCR} (n=10) or MLL-AF6^{RA1} (n=9) expressing cells induce rapid and invasive myeloid leukemias that spread to the spleen and thymus in secondary (not shown) and tertiary transplantation. Illustrated are thymi and spleen from mice transplanted each with $2x10^5$ bone marrow cells from secondary recipients. (**h**) Survival curves for mice transplanted with the MLL-AF6^{NCR} or MLL-AF6^{RA1} retroviral transduced cells from secondary recipients (panel **g**). Error bars are s.d.

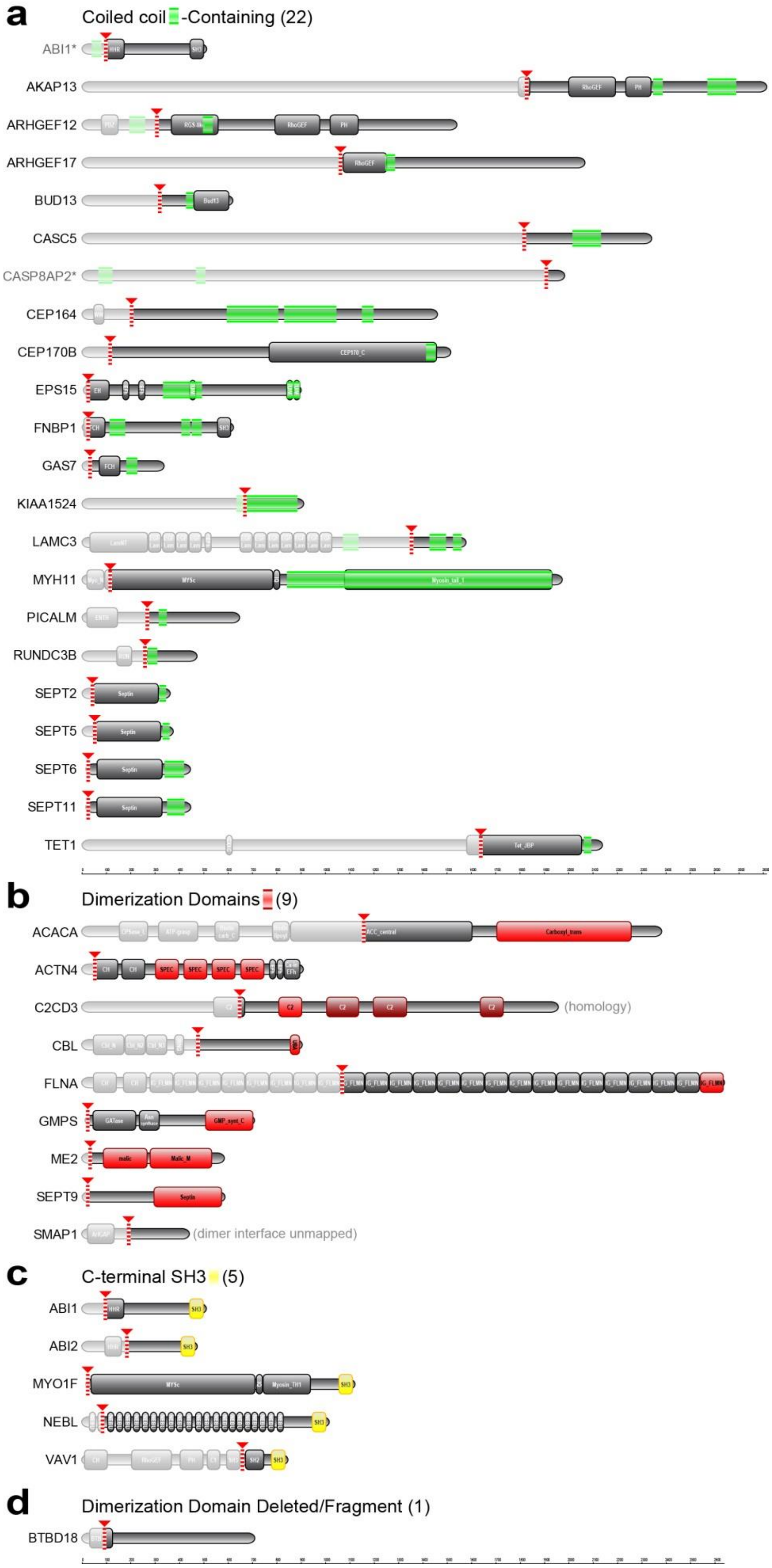
a
Coiled coil -Containing (22)
ABI1*
AKAP13
ARHGEF12
ARHGEF17
BUD13
CASC5
CASP8AP2*
CEP164
CEP170B
EPS15
FNBP1
GAS7
KIAA1524
LAMC3
MYH11
PICALM
RUNDC3B
SEPT2
SEPT5
SEPT6
SEPT11
TET1
b
Dimerization Domains (9)
ACACA
ACTN4
C2CD3
(homology)
CBL
FLNA
GMPS
ME2
SEPT9
SMAP1
(dimer interface unmapped)
c
C-terminal SH3 (5)
ABI1
ABI2
MYO1F
NEBL
VAV1
d
Dimerization Domain Deleted/Fragment (1)
BTBD18

Supplementary Fig. 6. Position of coiled coil or dimerization domains in relation to *MLL* breakpoints in protein partners of cytoplasmic origin. (**a**) 22 fusion proteins predicted to encode coiled coils (green; LOGICOIL and MARCOIL, 90% confidence). Fusion points with MLL are marked in red (dashed, arrow). Only in ABI1 and CASP8AP2 does the predicted coiled coil segment precede the breakpoint (*). (**b**) 9 fusion proteins are not predicted to have coiled coils, but dimerize by alternative mechanisms (red). (**c**) 5 fusion proteins encode C-terminal SH3 domains (yellow), related to SH3 domains known to dimerize. (**d**) MLL fusion in the final cytoplasmic protein partner (BTBD18) occurs within a BTB domain, analogous to the breakpoint within the RA1 domain of AF6.

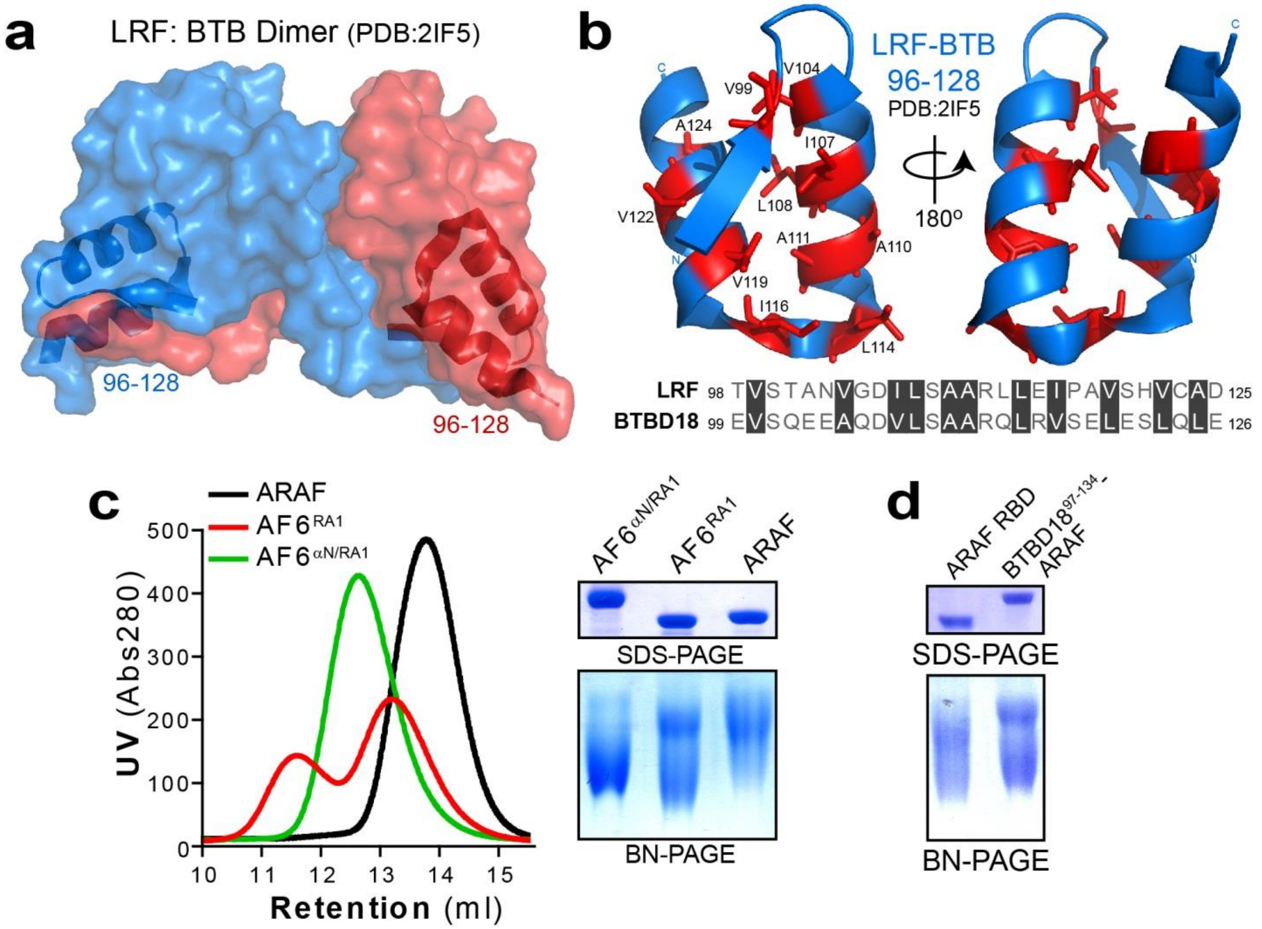

Supplementary Fig. 7. Dimerization of MLL induced by exposed hydrophobic interfaces in partner domains is conserved in BTBD18. (**a**) Fusion of the *BTBD18* gene to *MLL* results in an MLL-BTBD18 fusion protein with a truncated BTB domain at the interface. BTB domains typically form dimers; however, the BTB dimerization interface in MLL-BTBD18 is lost. A BTB domain dimer from LRF (PDB 2IF5) is shown to demonstrate the truncated fragment resulting from fusion with MLL. Surface representation shows two individual BTB domains that interact to form a dimer (red, blue). Just two small helices remain at the MLL fusion interface, corresponding to residues 96-128 in LRF (ribbons). (**b**) The remaining BTB domain fragment at the junction with MLL following its fusion. Ribbons model is based on a BTB domain from the highly related LRF (PDB 2IF5; sequence similarity at bottom). The resulting helix-turn-helix presents one completely hydrophobic interface (red). (**c**) Left, size exclusion chromatography establishes the ARAF RBD domain as a monomer, in comparison with the AF6 RA1 domain which is a dimer in the absence of the αN helix ($AF6^{37\text{-}136}$), but a monomer when it is included ($AF6^{6\text{-}136}$). Right, these data are corroborated by BN-PAGE analysis. (**d**) BN-PAGE oligomerization analysis following fusion of the MLL-generated helix-turn-helix from BTBD18 to the normally monomeric ARAF RBD domain.

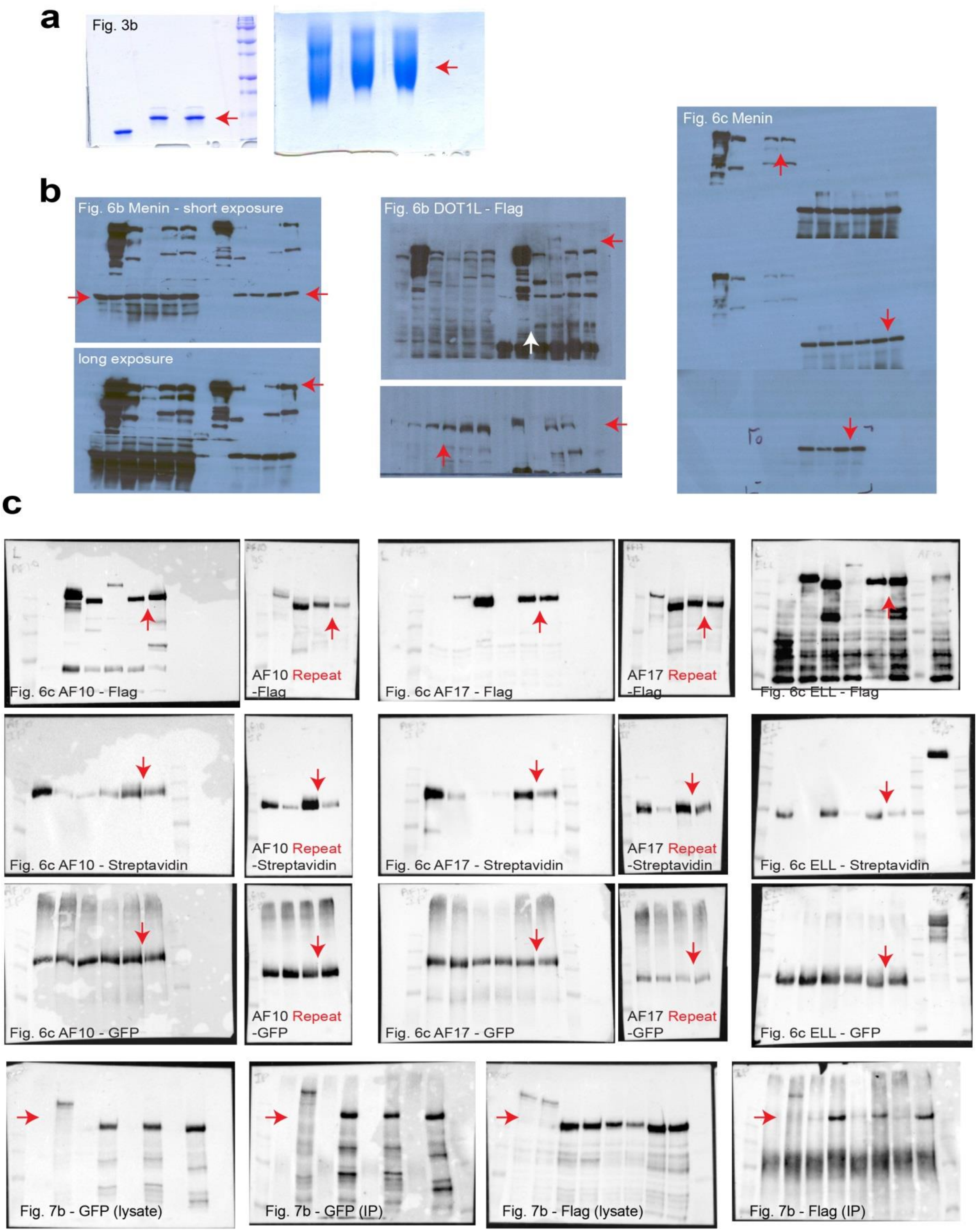
a
Fig. 3b
b
Fig. 6b Menin - short exposure
long exposure
Fig. 6b DOT1L - Flag
Fig. 6c Menin
c
Fig. 6c AF10 - Flag
AF10 Repeat -Flag
Fig. 6c AF17 - Flag
AF17 Repeat -Flag
Fig. 6c ELL - Flag
Fig. 6c AF10 - Streptavidin
AF10 Repeat -Streptavidin
Fig. 6c AF17 - Streptavidin
AF17 Repeat -Streptavidin
Fig. 6c ELL - Streptavidin
Fig. 6c AF10 - GFP
AF10 Repeat -GFP
Fig. 6c AF17 - GFP
AF17 Repeat -GFP
Fig. 6c ELL - GFP
Fig. 7b - GFP (lysate)
Fig. 7b - GFP (IP)
Fig. 7b - Flag (lysate)
Fig. 7b - Flag (IP)

Supplementary Fig. 8. Uncropped images of gels and Western blots. (**a)** Coomassie-stained SDS-PAGE and BN-PAGE gels from Fig. 3b. (**b**) Western blots from Fig. 6b and Fig. 6c; chemiluminescent detection was done with X-Ray film and a developer. (**c**) Western blots from Fig. 6c and 7b; chemiluminescent detection was done with a ChemiDoc imager. Blots are overlaid with an image taken under white light to display the nitrocellulose membrane and the position of MW markers.

Supplementary Data Files

Supplementary Data 1 (Excel)**.** BioID protein interactors for the MLL N-terminus alone (MLLN), MLLN fused to the AF6 RA1 domain (RA1, dimer), or MLL fused to the AF6 RA1 domain with extended-αN helix (αN/RA1, monomer). Post-SAINT SpecSum lists peptide counts for each interactor following SAINT analysis (iProphet probability of 0.95 and data filtered with an FDR>0.02). Highest confidence prey's (FDR>0.01) are shaded grey.

Supplementary Data 2 (Excel)**.** Annotation for expanded interactome of BioID partner proteins specific for baits MLLN and monomeric MLL-αN/RA1, or for dimeric MLL-RA1. Enrichment was done with iRefIndex (IntAct, BioGRID, MINT, and DIP). Data was input to Cytoscape for assembly of the interactome in Fig. 6a.

Supplementary Table 1. Potential coiled coils in 58 gene partners from the *MLL* recombinome. 20 partner genes localized to the nucleus were omitted from study, as they may possess alternative mechanisms of transcriptional activation, and 2 genes fused out of frame to *MLL* were also excluded. The remaining 36 fused genes encode cytoplasmic proteins, and coiled coil predictions were used to determine their propensity to oligomerize (LOGICOIL and MARCOIL, 90% confidence). For the 22 partner proteins with coiled coils, their positon in relation to the MLL fusion is further depicted in Supplementary Fig. 6a.

	**Gene Name**	**Entrez ID**	**Location**	**GO CC**	**# aa**	**# cc**	**LOGICOIL**	**MARCOIL 90% Confidence cc Region**	**Break**
**22**	ABI1	10006	10p12.1	Cytosol	508	1	Parallel Dimer	39-76	96
	AKAP13	11214	15q24-q25	Cytosol	2817	2	Tetramer	2352-2383, 2579-2679	1827
	ARHGEF12	23365	11q23.3	Cytosol	1544	3	Antiparallel Dimer	205-225, 235-254, 496-537	309
	ARHGEF17	9828	11q13.3	Cytosol	2063	(1)	Antiparallel Dimer	-	1065
	BUD13	84811	11q23.3	Cytosol	619	1	Antiparallel Dimer	437-485	347
	CASC5	57082	15q14	Cytosol	2342	2	Parallel Dimer	2024-2065, 2074-2129	1819
	CASP8AP2	9994	6q15	Cytosol	1982	2	Parallel Dimer-Tetramer	72-122, 470-513	1932
	CEP164	22897	11q23.3	Cytosol	1460	3	Trimer-Tetramer	599-804, 837-1043, 1159-1196	256
	CEP170B	283638	14q32.33	Cytosol	1519	1	Parallel Dimer	1421-1434	112
	EPS15	2060	1p32	Cytosol	896	2	Tetramer	334-498, 867-880	12
	FNBP1	23048	9q34	Cytosol	617	3	Tetramer	106-185, 411-433, 459-480	9
	GAS7	8522	17p13.1	Cytosol	336	1	Tetramer	188-220	36
	KIAA1524	57650	3q13.13	Cytosol	905	2	Parallel Dimer	630-710, 714-878	671
	LAMC3	10319	9q31-q34	Cytosol	1575	5	Tetramer	1079-1130, 1206-1229, 1274-1281, 1283-1343, 1369-1489	1345
	MYH11	4629	16p13.11	Cytosol	1972	4	Dimer	851-1188, 1192-1575, 1579-1805, 1816-1927	116
	PICALM	8301	11q14	Cytosol	652	1	Parallel Dimer	326-340	256
	RUNDC3B	154661	7q21.12	Cytosol	473	1	Tetramer	281-326	284
	SEPT11	55752	4q21.1	Cytosol	429	1	Tetramer	351-410	20
	SEPT2	4735	2q37.3	Cytosol	361	1	Tetramer	320-342	45
	SEPT5	5413	Xq24	Cytosol	369	1	Parallel Dimer	350-364	61
	SEPT6	23157	22q11.2	Cytosol	429	1	Parallel Dimer	348-399	11
	TET1	80312	10q21	Cytosol	2136	1	Tetramer	2070-2090	1639
**14**	ABI2	10152	2q33	Cytosol	475	0	-	-	
	ACACA	31	17q21	Cytosol	2383	0	-	-	
	ACTN4	81	19q13	Cytosol	911	2	Parallel Dimer (In SPEC)	450-480, 684-710	
	BTBD18	643376	11q12.1	Cytosol	712	0	-	-	
	C2CD3	26005	11q13.4	Cytosol	1963	0	-	-	
	CBL	867	11q23.3	Cytosol	906	0	-	-	
	FLNA	2316	Xq28	Cytosol	2639	0	-	-	
	GMPS	8833	3q25.31	Cytosol	693	0	-	-	
	ME2	4200	18q21	Cytosol	584	0	-	-	
	MYO1F	4542	19p13.3-p13.2	Cytosol	1098	0	-	-	
	NEBL	10529	10p12	Cytosol	1014	0	-	-	
	SEPT9	10801	17q25.3	Cytosol	586	0	-	-	
	SMAP1	60682	6q12-q13	Cytosol	467	0	-	-	
	VAV1	7409	19p13.2	Cytosol	845	0	-	-	

Supplementary Table 2. Prediction of coiled coil regions in 40 randomly generated protein sequences (using the RSAT random gene generator). 61% of cytosolic proteins encoded by genes in *MLL* translocations are predicted to contain coiled coils, but only 12.5% of the randomly generated set. In terms of amino acid sequence coverage, 7.6 % of the MLL fused proteins are predicted to have coiled coils, but only 1.4% of the random set. Altogether, there is a 5-fold enrichment of coiled coils in cytosolic MLL partner proteins.

Total	Gene Name	Entrez ID	aa	cc	LOGICOIL	MARCOIL 90% Confidence cc Region
40	ALCF	29974	586	0	-	-
	CACYBP	27101	185	0	-	-
	CCDC153	283152	210	2	Tetramer	41-78, 98-147
	CDK14	5218	451	0	-	-
	CIB3	117286	187	0	-	-
	CLASP1	23332	1479	1	Tetramer	1249-1261
	CLEC1B	51266	196	0	-	-
	CNTNAP3B	79937	1288	0	-	-
	DDX24	57062	859	0	-	-
	DLG2	1740	975	0	-	-
	DOCK8	81704	2099	0	-	-
	DVL3	1857	716	0	-	-
	ELMO2	63916	720	0	-	-
	EMR2	30817	823	0	-	-
	FMNL3	91010	1027	3	Tetramer - Anti/parallel Dimer	395-418, 423-462, 931-952
	GART	2618	1010	0	-	-
	GRAP	10750	217	0	-	-
	HOXA5	3202	270	0	-	-
	IGFBP3	3486	291	0	-	-
	IL1RAPL2	26280	686	0	-	-
	IL5RA	3568	420	0	-	-
	IMPA2	3613	288	0	-	-
	KIF17	57576	1028	2	Tetramer - Antiparallel Dimer	411-444, 803-863
	KLHL10	317719	608	0	-	-
	METTL5	29081	209	0	-	-
	MTO1	25821	732	0	-	-
	NEDD9	4739	834	0	-	-
	NEUROG1	4762	237	0	-	-
	NRBP2	340371	501	0	-	-
	NXT2	55916	197	0	-	-
	OR7G3	390883	312	0	-	-
	PDE4A	5141	886	0	-	-
	POLM	27434	494	0	-	-
	PSPC1	55269	523	1	Antiparallel Dimer	283-371
	PTPRT	11122	1441	0	-	-
	RFX2	5990	723	0	-	-
	TBL1Y	90665	522	0	-	-
	TNFRSF19	55504	423	0	-	-
	ZBTB7C	201501	619	0	-	-
	ZNF527	84503	609	0	-	-

Random		
**Total:**	40	
**with cc (90%):**	5	
**%:**	12.5%	
**total cc (90%):**	9	
**% sequence coverage (90%):**	1.4%	
**MLL Cytosolic Partners**		
**Total:**	36	
**with cc (90%):**	22	**Difference**
**%:**	61.1%	4.9 X
**total cc (90%):**	44	4.9 X
**% sequence coverage (90%):**	7.6%	5.4 X

Supplementary Table 3. Dimerization of 15 cytosolic MLL partner proteins not predicted to contain coiled coils. MLL fusion occurs before known dimerization domains in 9 of these proteins, before a C-terminal SH3 domain in 5 partner proteins, and directly within a modular domain in 1 protein (BTBD18). The position of *MLL* breakpoints is further depicted in Supplementary Fig. 6b/c[1–19].

**15**	**9**	breakpoint|Recognized Dimerization Domain
	**5**	breakpoint|C-terminal SH3 domain
	**1**	breakpoint within BTB domain

Total		Gene	Domain	Boundary	Dimerizing	Break point	Fusion aa	Retained	Reference
**15**	**9**	ACACA	BC	58-570		Intron 14	158		
			BCCP	610-770				X	
			**CT**	**1484-2233**	**X**			**X**	Zhang et al. (2003) *Science*, 299, 2064–7.
		ACTN4	CH	52-152		Intron 1	55	X	Sjöblom et al. (2008) *Cellular and Molecular Life Sciences*, 65, 2688–2701.
			CH	165-264				X	
			**SPEC**	**296-402**	**X**			**X**	
			**SPEC**	**416-517**	**X**			**X**	
			**SPEC**	**531-638**	**X**			**X**	
			**SPEC**	**652-751**	**X**			**X**	
			EFh	769-797				X	
			EFh	810-838				X	
			EFh-CaIns	841-907				X	
		C2CD3	C2	539-677	X*	Intron 16	655		*2 closest human homologues by domain organization are dimers:*
			**C2**	**806-915**	**X***			**X**	Chapman et al. (1996) *Journal of Biological Chemistry*, 271(10), 5844–5849.
			**C2**	**1005-1145**	**X***			**X**	Xu et al. (2011) *PLoS ONE*, 6(11).
			**C2**	**1198-1337**	**X***			**X**	
			**C2**	**1636-1743**	**X***			**X**	
		CBL	CBL_N	49-176		Intron 9	478		
			CBL_N2	178-262					
			SH2	256-352					
			RING	381-423					
			**UBA**	**857-896**	**X**			**X**	Kozlov et al. (2007) *Journal of Biological Chemistry*, 282(37), 27547–27555.
		FLNA	CH	44-148		Intron 19	1071		
			CH	168-264					
			IG_FLMN	281-375					
			IG_FLMN	381-477					
			IG_FLMN	480-571					
			IG_FLMN	577-659					
			IG_FLMN	672-766					
			IG_FLMN	769-869					
			IG_FLMN	872-968					
			IG_FLMN	972-1064					
			IG_FLMN	1067-1156					
			IG_FLMN	1160-1252				X	
			IG_FLMN	1256-1352				X	
			IG_FLMN	1355-1442				X	
			IG_FLMN	1448-1542				X	
			IG_FLMN	1545-1639				X	
			IG_FLMN	1642-1735				X	
			IG_FLMN	1789-1853				X	
			IG_FLMN	1860-1946				X	
			IG_FLMN	2039-2128				X	
			IG_FLMN	2230-2320				X	
			IG_FLMN	2324-2415				X	
			IG_FLMN	2421-2511				X	
			**IG_FLMN**	**2549-2638**	**X**			**X**	Nakamura et al. (2007) *Journal of Cell Biology*, 179(5), 1011–1025.
		GMPS	GMP_SYNTH	28-207		Intron 1	10	X	
			**GMP_SYNTH_C**	**238-692**	**X**			**X**	Welin et al. (2013) *Journal of Molecular Biology*, 425(22), 4323–4333.
		ME2	**MALIC**	**89-270**	**X**	Intron 1	37	**X**	Murugan et al. (2012) *PLoS ONE*, 7(12), 1–11.
			**NAD_bind**	**280-558**	**X**			**X**	
		SEPT9	MCLC	82-256		Intron 3	25		
			**SEPTIN**	**295-583**	**X**			**X**	Sellin et al. (2011) Molecular Biology of the Cell, 22, 3152–3164.
		SMAP1	ARFGAP	19-136		Intron 6	193		*Unmapped dimer:* Natsume et al. (2006)Molecular Biology of the Cell, 17(June), 1018–1032.
	**5**	ABI1	SNARE	54-95		Intron 2	96		
			HHR	95-171				X	
			**SH3**	**449-504**				**X**	
		ABI2	SNARE	60-94		Intron 4	161		
			HHR	94-172				X	*Dimerization of related SH3 domains:*

			Biotinyl_lipoyl	268-317				X	Fan et al. (2003) *Cancer Research*, 63, 873–877.
			**SH3**	**416-471**				**X**	Levinson et al. (2009) *PLoS ONE*, 4(11), 1–5.
		MYO1F	MYSc_TYPE_1	18-689		Intron 1	2	X	Cámara-Artigas et al. (2009) *FEBS Letters*, 583(4), 749–753
			MYOSIN_TH1	716-918				X	Kishan et al. (2001) *Protein Science*, 10, 1046–1055.
			**SH3**	**1045-1097**				**X**	Kristensen et al. (2006) *The EMBO Journal*, 25(4), 785–797.
		NEBL	NEBU (23)	30-827		Intron 3	87	X	Ross et al. (2007) *Biochemical and Biophysical Research Communications*, 353, 463–468.
			**SH3**	**957-1014**				**X**	Harkiolaki et al. (2006) *Structure*, 14, 1741–1753.
		VAV1	CH	3-115		Intron 21	661		Romir et al. (2007) *Journal of Molecular Biology*, 365, 1417–1428.
			RHOGEF	198-372					Musacchio et al. (1994) *Nature Structural Biology*, 1, 546–551.
			PH	403-506					
			C1	516-564					
			SH3	595-659					
			SH2	669-751				X	
			**SH3**	**785-841**				**X**	
	**1**	BTBD18	BTB/POZ	34-132	X	Exon 2	98		

Supplementary References

1. Zhang, H., Zhiru, Y., Shen, Y. & Tong, L. Crystal structure of the carboxyltransferase domain of acetyl coenzyme A carboxylase. *Science (80-.).* **299,** 2064–7 (2003).
2. Sjöblom, B., Salmazo, a. & Djinović-Carugo, K. α-Actinin structure and regulation. *Cell. Mol. Life Sci.* **65,** 2688–2701 (2008).
3. Chapman, E. R., An, S., Edwardson, J. M. & Jahn, R. A Novel Function for the Second C2 Domain of Synaptotagmin: Ca-TRIGGERED DIMERIZATION. *J. Biol. Chem.* **271,** 5844–5849 (1996).
4. Xu, L. *et al.* Dysferlin forms a dimer mediated by the C2 domains and the transmembrane domain in vitro and in living cells. *PLoS One* **6,** (2011).
5. Kozlov, G. *et al.* Structural basis for UBA-mediated dimerization of c-Cbl ubiquitin ligase. *J. Biol. Chem.* **282,** 27547–27555 (2007).
6. Nakamura, F., Osborn, T. M., Hartemink, C. a., Hartwig, J. H. & Stossel, T. P. Structural basis of filamin A functions. *J. Cell Biol.* **179,** 1011–1025 (2007).
7. Welin, M. *et al.* Substrate specificity and oligomerization of human GMP synthetase. *J. Mol. Biol.* **425,** 4323–4333 (2013).
8. Murugan, S. & Hung, H. C. Biophysical Characterization of the Dimer and Tetramer Interface Interactions of the Human Cytosolic Malic Enzyme. *PLoS One* **7,** 1–11 (2012).
9. Sellin, M. E., Sandblad, L., Stenmark, S. & Gullberg, M. Deciphering the rules governing assembly order of mammalian septin complexes. *Mol. Biol. Cell* **22,** 3152–3164 (2011).
10. Natsume, W. *et al.* SMAP2, a Novel ARF GTPase-activating Protein, Interacts with Clathrin and Clathrin Assembly Protein and Functions on the AP-1–positive Early Endosome/Trans-Golgi Network. *Mol. Biol. Cell* **17,** 1018–1032 (2006).
11. Fan, P., Cong, F. & Goff, S. P. Homo- and Hetero-Oligomerization of the c-Abl Kinase and Abelson-Interactor-1. *Cancer Res.* **63,** 873–877 (2003).
12. Levinson, N. M., Visperas, P. R. & Kuriyan, J. The tyrosine kinase Csk dimerizes through its SH3 domain. *PLoS One* **4,** 1–5 (2009).
13. Cámara-Artigas, A., Martín-García, J. M., Morel, B., Ruiz-Sanz, J. & Luque, I. Intertwined dimeric structure for the SH3 domain of the c-Src tyrosine kinase induced by polyethylene glycol binding. *FEBS Lett.* **583,** 749–753 (2009).
14. Kishan, K. V, Newcomer, M. E., Rhodes, T. H. & Guilliot, S. D. Effect of pH and salt bridges on structural assembly: molecular structures of the monomer and intertwined dimer of the Eps8 SH3 domain. *Protein Sci.* **10,** 1046–1055 (2001).
15. Kristensen, O. *et al.* A unique set of SH3-SH3 interactions controls IB1 homodimerization. *EMBO J.* **25,** 785–797 (2006).
16. Ross, B. *et al.* High resolution crystal structures of the p120 RasGAP SH3 domain. *Biochem. Biophys. Res. Commun.* **353,** 463–468 (2007).
17. Harkiolaki, M., Gilbert, R. J. C., Jones, E. Y. & Feller, S. M. The C-Terminal SH3 Domain of CRKL as a Dynamic Dimerization Module Transiently Exposing a Nuclear Export Signal. *Structure* **14,** 1741–1753 (2006).
18. Romir, J. *et al.* Crystal Structure Analysis and Solution Studies of Human Lck-SH3; Zinc-induced Homodimerization Competes with the Binding of Proline-rich Motifs. *J. Mol.*

Biol. **365,** 1417–1428 (2007).

19. Musacchio, a, Saraste, M. & Wilmanns, M. High-resolution crystal structures of tyrosine kinase SH3 domains complexed with proline-rich peptides. *Nat. Struct. Biol.* **1,** 546–551 (1994).
