# Supplementary material for: Evolution of AF6-RAS association and its implications in mixed-lineage leukemia
Source: Nat Commun. 2017 Oct 23;8:1099. doi: 10.1038/s41467-017-01326-5 (PMC5653649; doi:10.1038/s41467-017-01326-5)
Supplement: Supplementary file 2 — Descriptions of Additional Supplementary Files [file 41467_2017_1326_MOESM2_ESM.pdf]

Description of Additional Supplementary Files

File Name: Supplementary Dataset 1
Description: BioID protein interactors for the MLL N-terminus alone (MLLN), MLLN fused to the AF6 RA1 domain (RA1, dimer), or MLL fused to the AF6 RA1 domain with extended-□N helix (□N/RA1, monomer). Post-SAINT SpecSum lists peptide counts for each interactor following SAINT analysis (iProphet probability of 0.95 and data filtered with an FDR>0.02). Highest confidence prey’s (FDR>0.01) are shaded grey.

File Name: Supplementary Dataset 2
Description: Annotation for expanded interactome of BioID partner proteins specific for baits MLLN and monomeric MLL-αN/RA1, or for dimeric MLL-RA1. Enrichment was done with iRefIndex (IntAct, BioGRID, MINT, and DIP). Data was input to Cytoscape for assembly of the interactome in Fig. 6a.
